# *Drosophila* Modulo is essential for transposon silencing and developmental robustness

**DOI:** 10.1016/j.jbc.2025.108210

**Published:** 2025-01-22

**Authors:** Rasesh Y. Parikh, Dhananjaya Nayak, Haifan Lin, Vamsi K. Gangaraju

**Affiliations:** 1 Department of Biochemistry and Molecular Biology and Hollings Cancer Center, Medical University of South Carolina, Charleston, South Carolina, USA; 2 Yale Stem Cell Center and Department of Cell Biology, Yale University, New Haven, Connecticut, USA

**Keywords:** canalization, developmental robustness, piwi, piRNA pathway, modulo

## Abstract

Transposable element (TE) silencing in the germline is crucial for preserving genome integrity; its absence results in sterility and diminished developmental robustness. The Piwi-interacting RNA (piRNA) pathway is the primary small non-coding RNA mechanism by which TEs are silenced in the germline. Three piRNA binding proteins promote the piRNA pathway function in the germline– P-element-induced wimpy testis (Piwi), Aubergine (Aub), and Argonaute 3 (Ago3). Piwi mediates transcriptional silencing of TEs by promoting the deposition of the heterochromatin mark Histone 3 lysine nine trimethylation (H3K9me3) at TE genomic sites. Aub and Ago3 facilitate post-transcriptional silencing of TEs. Proteins and mechanisms that promote piRNA function in TE silencing are still being discovered. This study demonstrates that the *Drosophila* Modulo protein, a homolog of mammalian Nucleolin and an epigenetic regulator, is crucial for the enrichment of H3K9me3 at TEs. We show that Modulo interacts with Piwi and operates downstream of the Piwi-piRNA complex's entry into the nucleus. Lack of Modulo function impairs Piwi-interacting protein Panoramix's ability to target transposon RNAs. Furthermore, the reduced function of Modulo in the mother undermines developmental robustness and exacerbates neomorphic *Kr[If-1]*-induced ectopic eye outgrowths in the offspring. Maternal Modulo enhances developmental robustness by inhibiting TE activation and transcriptome variability associated with intrinsic genetic variation. Thus, Modulo is an essential component of the mechanism that operates in the maternal germline to facilitate TE silencing and ensure developmental robustness in the ensuing generation.

Transposons are selfish genetic elements that can independently replicate their sequences and cause chaos in the genome (1). The prevention of transposon activity is a priority for the organism, especially in the germline, as the mutations caused by uncontrolled transposon movement can be inherited in later generations. Transposons are highly prevalent in the genomes of higher organisms, occupying 12% and 45% of *Drosophila* and human genomes, respectively (2, 3). Defective transposon silencing can create *de novo* mutations (4) or affect the expression of neighboring genes (5). Distinct small non-coding RNA pathways silence transposons in the soma and germline. The endogenous short-interfering RNA (siRNA) pathway silences transposons in the somatic tissues (6). The Piwi-interacting RNA (piRNA) pathway specializes in silencing transposons in the germline (7, 8, 9). In *Drosophila*, the primary function of the piRNA pathway is mediated by three piRNA binding proteins – P-element induced wimpy testis (Piwi), Aubergine (Aub), and Argonaute 3 (Ago3) (7). All three proteins use the sequence of the bound ∼26 nucleotide(nt)-long piRNAs as the guide and target complementary sequences in the transposon transcripts. Inside the nucleus, Piwi, in collaboration with Panoramix (Panx), and several other proteins, promotes the deposition of histone H3 lysine nine trimethylation (H3K9me3) mark at the transposon genomic sites and prevents transcription at these sites (10, 11, 12, 13, 14, 15, 16, 17, 18, 19, 20, 21).

Positive selection fixes phenotypes that provide a fitness advantage to organisms, and canalization (22), also known as developmental robustness, maintains the fidelity of these phenotypes despite inherent genetic variations and environmental perturbations (23, 24, 25, 26, 27). Decanalization and the phenotypic manifestation of cryptic variations allow organisms to adapt to new environments. Earlier work from Susan Lindquist’s group showed that mutations in the protein chaperone Heat shock protein 90 (Hsp90) release cryptic variants that can then be fixed in a population independent of the Hsp90 mutation (28), thus providing a molecular mechanism for the concept first conceived by Conrad Waddington in the 1950s (22). Since then, Hsp90 has been shown to buffer genetic variations in many organisms (29, 30, 31) *via* multiple mechanisms (4, 5, 28, 30, 32, 33).

Hsp90’s influence on the piRNA pathway and transposon activity intertwined canalization and transposon silencing fields. One of the first indications that transposon silencing is involved in canalization came from the study that showed that the piRNA pathway protein Piwi promotes developmental robustness (33). A reduction in the dosage of Piwi in the mother but not the father induced ectopic eye outgrowths (EEO) in the progeny in the presence of a neomorphic allele of the transcription factor Krüppel, *Kr*^*If-1*^. Piwi function in the maternal germline promotes canalization; however, the biochemical mechanism is unknown. This study shows that the chromatin-associated factor Modulo (Mod) (34, 35) interacts with Piwi in ovaries and is needed for the epigenetic silencing of transposons and developmental robustness. Lack of Mod leads to a drastic decrease in the H3K9me3 enrichment at transposon genomic sites with a concomitant increase in the steady-state transposon mRNA levels. Mod is essential for Panoramix’s ability to interact with target transposon RNAs, a critical step in the transcriptional gene silencing of transposons. We finally demonstrate that the Mod function in the ovary promotes the developmental robustness of subsequent generations by preventing inherent genetic variation-induced transposon activation and transcriptome variance.

## Results

**Figure 1:**
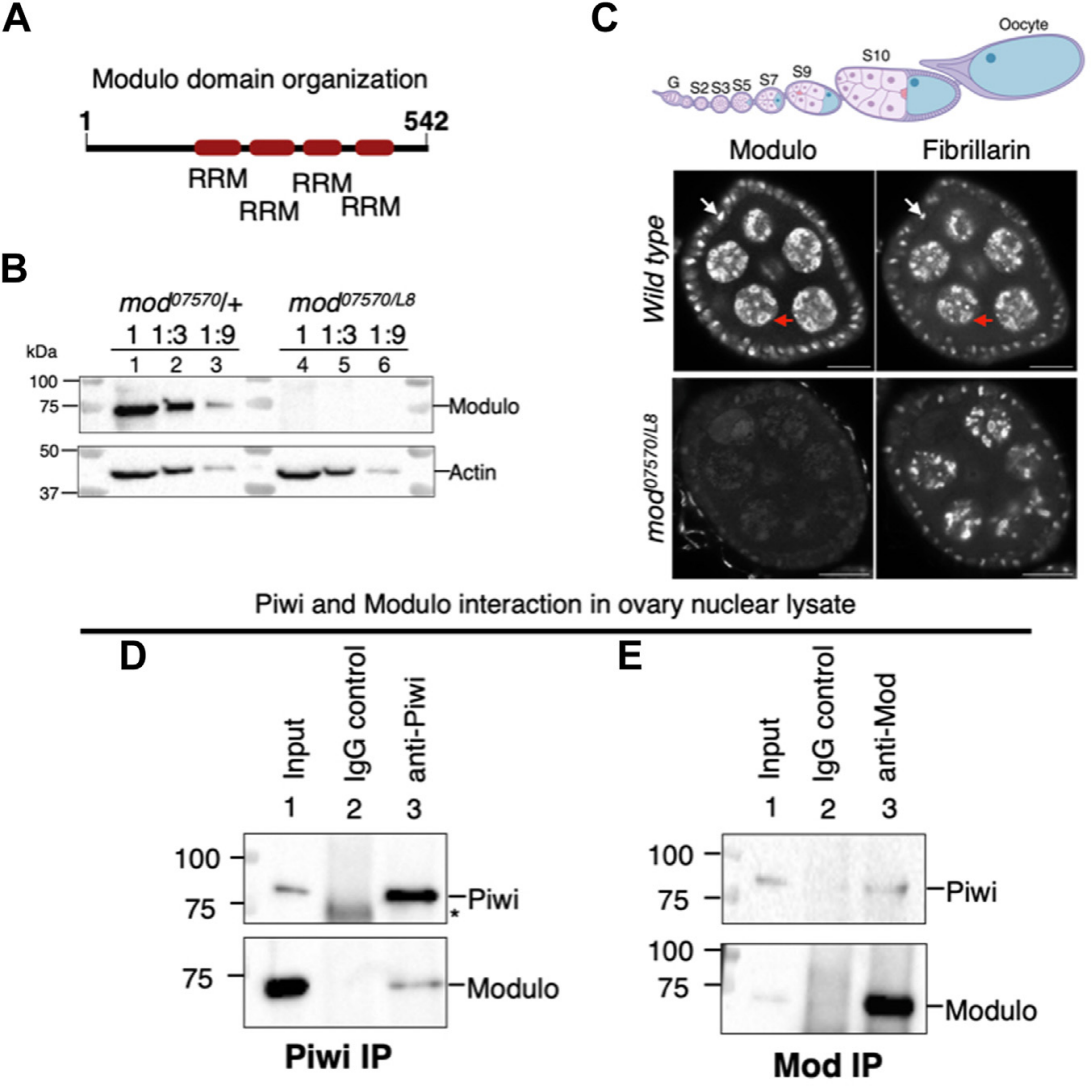
**Modulo interacts with Piwi.***A*, the Domain organization of Modulo. Please note that the image is not drawn to scale. RRM stands for RNA recognition motif. *B*, immunoblot analysis to test Mod protein levels using a Mod antibody generated in this study. A transheterozygous combination of *mod* alleles (*mod*^*07570/L8*^) depletes Modulo protein from the ovaries compared to the heterozygous control, *mod*^*07570*^/+. Ratios represent a three-fold serial dilution of ovary lysates. *C*, *Drosophila* ovary egg chambers costained for Mod and Fibrillarin in wild type control (*Oregon-R*) and *mod*^*07570/L8*^. *White* and *red* arrows mark somatic and germ cells, respectively. Scale bar, 10 μm. On *top* is a schematic of an ovariole with several developmental stages of egg chambers. G stands for germarium. The illustration was created in BioRender. *D* and *E*, immunoblot analysis of Piwi immunoprecipitation (IP) (*C*) and Mod IP (*D*) assessing Piwi-Modulo interaction in *Oregon-R* ovary lysates. Please compare lane 3 with the control lane 2 in C and D. ∗ represents a non-specific band recognized by Piwi antibody.

We followed a column chromatography scheme to purify Piwi-interacting proteins from a 0 to 12 h embryo extract to gain insights into the biochemical mechanism by which Piwi silences transposons and promotes developmental robustness (33). This strategy previously showed that the co-chaperone Stip1 and the nuclear pore protein Nup358 interact with Piwi (33, 36, 37). In addition to Stip1, and Nup358, we have identified the chromatin-associated protein Modulo (Mod) comigrating with Piwi. Mod is a homolog of mammalian Nucleolin and contains four RNA recognition motifs (RRM) (34) (Fig. 1*A*). While RRMs allow Mod to bind RNA in a sequence-specific manner, positively charged amino acid stretches in the N- and C-termini enable it to bind DNA with no sequence specificity (38). Mod has been shown to function downstream of pattern-forming genes and is necessary for proper morphogenesis (34, 35). *mod* deletions are recessive lethal and act as dominant suppressors of position effect variation (PEV) (34). These data suggest that Mod is involved in chromatin compaction and silencing processes. We decided to characterize Mod's potential involvement in Piwi function because of its co-migration with Piwi on column chromatography and its demonstrated role in epigenetic regulation. To study Mod function, we used a transheterozygotic mutant, *mod*^*07570/L8*^. *mod*^*07570*^ is a hypomorphic allele generated by a P-element insertion (39). *mod*^*L8*^ is an amorphic allele generated by a subterminal deletion in chromosome 3R (34). Both *mod*^*07570*^ and *mod*^*L8*^ are recessive lethal; however, transheterozygous *mod*^*07570/L8*^ flies survive and are fertile. Mod protein levels are greatly diminished in *mod*^*07570/L8*^ compared to the heterozygous control, *mod*^*07570*^*/+* (Figs. 1*B* and S1*A*, compare lanes 4–6 with 1–3). Consistent with this, upon confocal microscopy, the Mod protein signal is depleted in *mod*^*07570/L8*^ ovaries, compared to the control (Fig. 1*C*, compare Mod panels in Wild type and *mod*^*07570/L8*^). Furthermore, in agreement with its nucleolar function (38, 40, 41), Mod colocalizes with Fibrillarin (Fib), a nucleolar marker (see red arrows in the germline and white arrows in the somatic cells in Fig. 1*C* upper panel). Reciprocal co-immunoprecipitations using anti-Piwi (Figs. 1*D* and S1*B*) and Mod antibodies (Figs. 1*E* and S1*C*) showed that Piwi and Mod interact in ovary nuclear lysates (Fig. 1, *D* and *E*, compare lanes 2 and 3).

**Figure 2:**
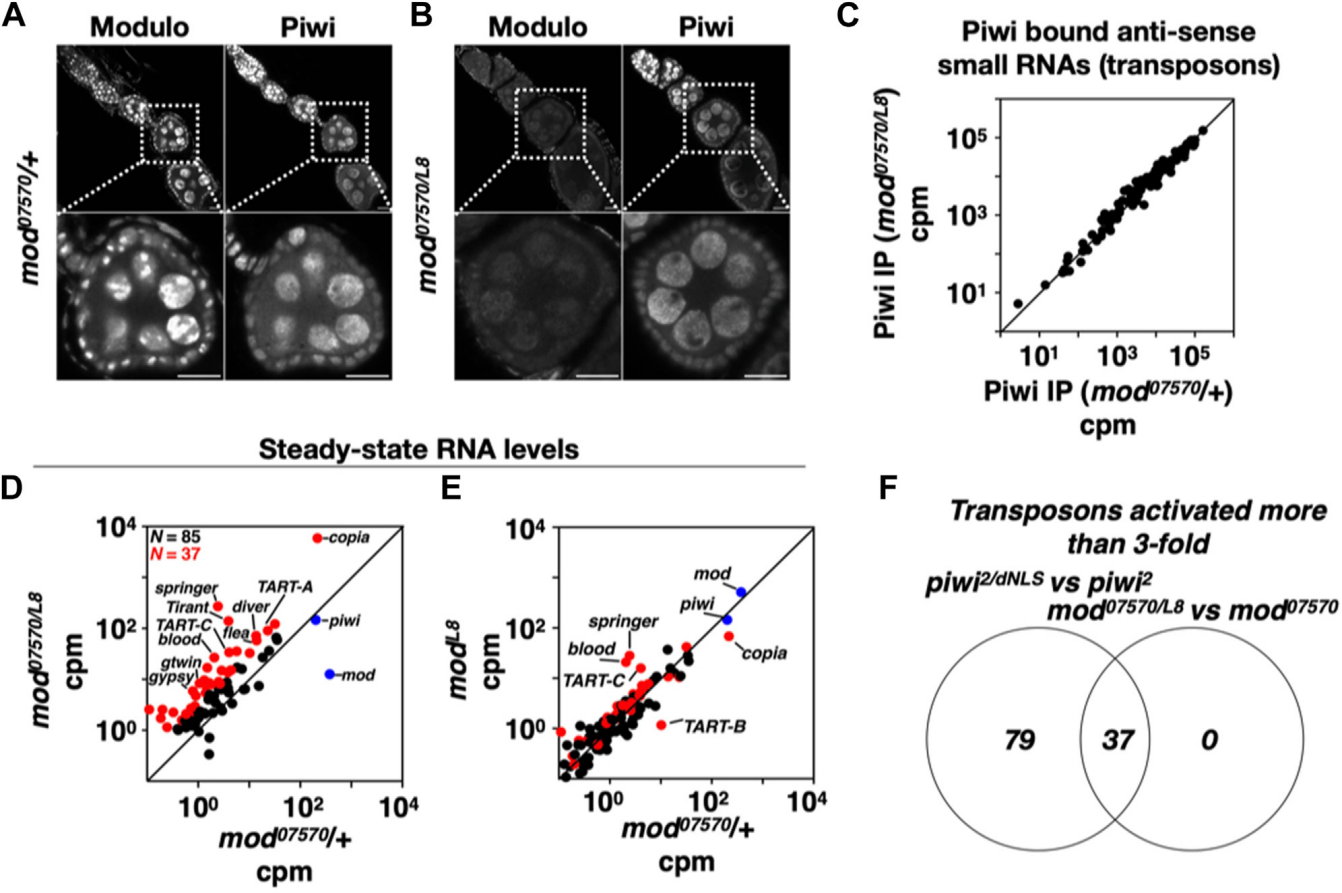
**Regulation of Piwi function and transposon silencing by Mod.***A* and *B*, confocal images of ovarioles (*top panels*) and select egg chambers (*bottom panels*) costained for Modulo (Mod) and Piwi in the indicated genotypes. *C*, XY scatter plot showing the levels of anti-transposon small RNAs (antisense) bound to Piwi in the indicated genotypes. Reads are normalized as counts per million (cpm) reads that map to the *dm3* genome. Results from one biological replicate are shown here. *D* and *E*, XY scatter plots showing the steady-state mRNA levels of transposons in various genotypes. The average of 3 biological replicates is plotted. Reads were normalized as cpm reads that map to transposon consensus sequences and Refseq genes. Data points in *red* mark transposons activated more than 3-fold in D. *F*, a Venn diagram comparing the number of transposons activated more than 3-fold in *piwi*^*2/dNLS*^ and *mod*^*07570/L8*^ ovaries. Please note that the size of the circles does not correspond to the size of the data sets they represent.

Next, we investigated if Mod regulates Piwi function. Piwi protein levels did not change in the absence of Mod, showing that Mod is dispensable for Piwi protein stability(Fig. S2*A*, compare lanes 5–8 with 1–4). Confocal microscopy has revealed that Mod is not needed for Piwi nuclear localization (Fig. 2, *A* and *B*, compare Piwi staining pattern). Moreover, in the absence of Mod, Piwi binding to anti-transposon small RNAs remains unaffected (Fig. 2*C*). Neither the size distribution of Piwi-bound piRNAs (Fig. S2*B*) nor their composition (Fig. S2*C*) changed in the absence of Modulo. These results show that Mod is dispensable for all the steps in the Piwi function leading up to the epigenetic silencing of transposons. To determine the necessity of Mod for transposon silencing, we assessed steady-state transposon mRNA levels in the trans heterozygous combination *mod*^*07570/L8*^ and the two heterozygous controls *mod*^*07570*^/+ and *mod*^*L8*^/+ (Fig. 2, *D* and *E*). Sequenced reads were normalized as counts per million mapped reads (cpm) following their mapping to RefSeq genes and transposons. Among the 85 transposons exhibiting a minimum of one cpm, 37 (∼43%) were activated over three times in *mod*^*07570/L8*^ ovaries relative to *mod*^*07570*^/+ (Fig. 2*D*, red data points). The comparable mRNA levels of the majority of these 37 transposons in the two heterozygous controls (Fig. 2*E*, red data points) indicate that transposon activation in *mod*^*07570/L8*^ ovaries results from the lack of Mod function rather than genetic background differences. The steady-state mRNA levels of a few transposons exhibited modest differences in *mod*^*07570*^/+ or *mod*^*L8*^/+ (Fig. 2*E*, indicated transposons); nevertheless, these were subsequently activated over threefold in *mod*^*07570/L8*^ ovaries. Consistent with no change in Piwi protein levels (Fig. S2*A*), Piwi mRNA transcript level did not change with the loss of Mod, confirming that transposon activation is not due to a decrease in *piwi* mRNA levels (Fig. 2*D*, blue data point). A comparison of *piwi* and *mod*-dependent transposon silencing revealed that all transposons triggered in *mod*^*07570/L8*^ ovaries were likewise activated in *piwi*^*2/dNLS*^ ovaries, where Piwi is nonfunctional (42) (Fig. 2*F*). Our analysis indicates that Modulo is essential for silencing approximately 32% of the transposons that are also silenced by Piwi (Fig. 2*F*).

**Figure 3:**
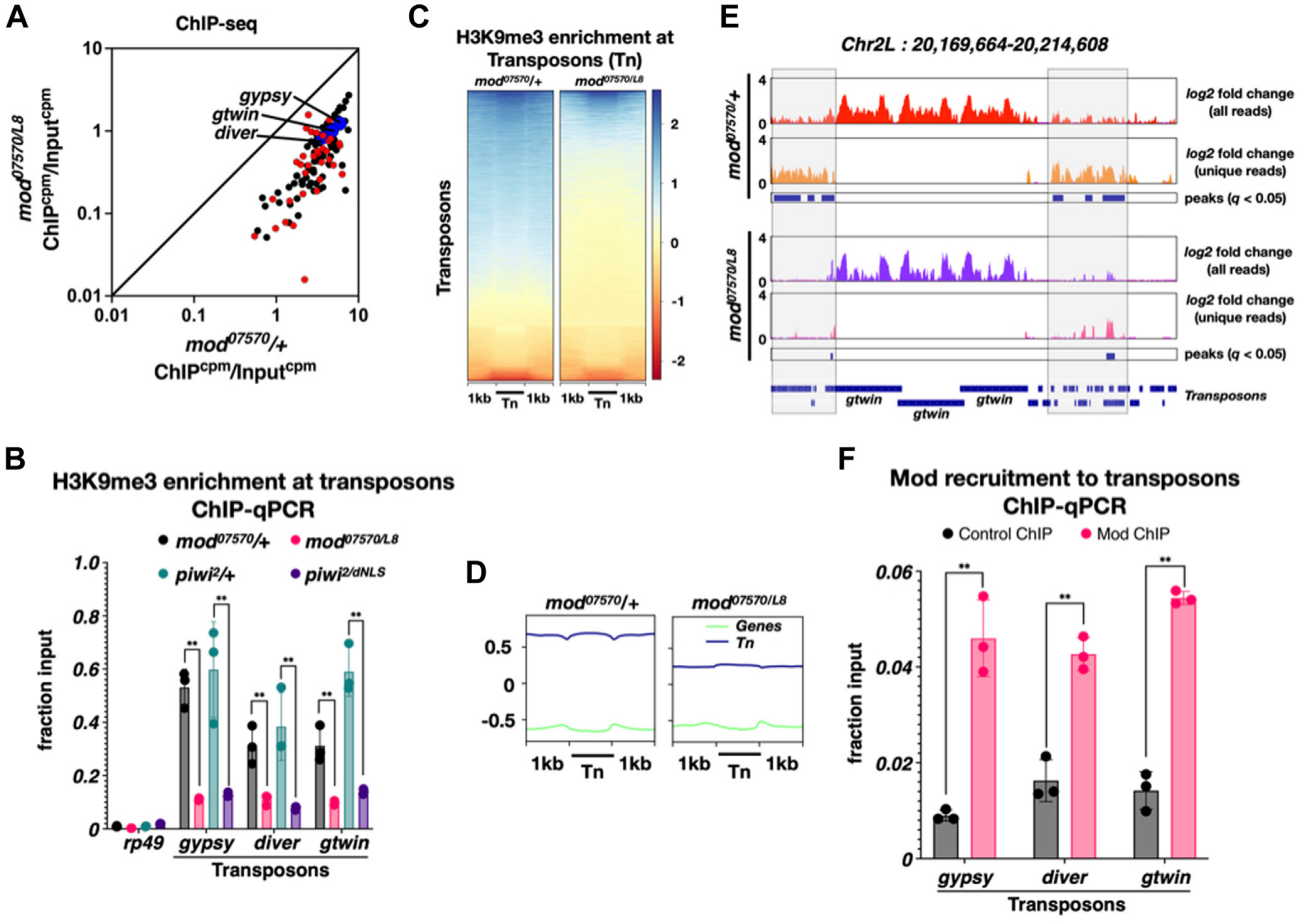
**Modulo is required for H3K9me3 enrichment at transposon genomic sites.***A*, a XY scatter plot showing the H3K9me3 enrichment (ChIP/Input) at various transposons in *mod*^*07570*^/+ and *mod*^*07570/L8*^ ovaries. Data points in *red* indicate transposons activated greater than 3-fold in Fig. 2*D*. The three transposons (*gypsy*, *diver*, and *gtwin*) that were further tested in 3B and F are marked in *blue*. The result from one biological replicate is shown here. Please see Fig 3*B* for further confirmation using ChIP-qPCR. *B*, a column plot showing the result for the ChIP-qPCR experiment testing H3K9me3 levels at three representative transposons and the *rp49* gene. Individual data points represent three independent biological replicates. The *p*-value was calculated using an unpaired *t* test. ∗∗ represents a *p*-value less than 0.01. *C*, a heat map derived from the result in Fig. 3*A*, representing H3K9me3 enrichment at various genomic locations of transposons and 1kb on either side of the transposon. The scale bar for fold-change is shown on the *right*. *D*, meta plots generated by *plotProfile* tool showing the enrichment of H3K9me3 along the gene body and 1kb on either side of transposon (Tn) genomic locations and Refseq genes. *E*, genome browser view of three *gtwin* insertions and surrounding regions on chromosome 2L. *log2* fold change in H3K9me3 enrichment as determined by the *bamCompare* tool is shown for all and unique reads. Shaded boxes show the location of peaks determined by the *MACS2* tool. Peaks were determined using the uniquely mapped reads. The *q-value* cutoff for the peaks was set at 0.05. Peaks are shown in *blue* bars. The genomic location of the three *gtwin* insertions is provided at the *bottom* of the figure. *F*, the results of the ChIP-qPCR experiment testing Mod recruitment are displayed in a column plot. ChIP using isotype IgG was used as a control. Individual data points represent three independent biological replicates. The *p*-value was calculated using an unpaired *t* test. ∗∗ represents a *p*-value less than 0.01.

Next, we examined if H3K9me3 enrichment at transposons gets affected in the absence of Mod. H3K9me3 levels at transposons were quantified with and without Mod through chromatin immunoprecipitation (ChIP) followed by sequencing. Sequenced reads from ChIP and input samples were aligned to RefSeq genes and transposons and normalized as counts per million mapped reads (cpm). The cpm in ChIP and input samples were compared to determine relative enrichment (ChIP^cpm^/Input^cpm^). The H3K9me3 enrichment at ∼98% (119 out of 122) transposons reduced by more than 3-fold in *mod*^*0757/L8*^ ovaries compared to the heterozygous control, *mod*^*07570*^/+(Fig. 3*A*). Furthermore, 92% of the transposons activated more than 3-fold in Fig. 2*D* exhibited a greater than 3-fold reduction in H3K9me3 levels (Fig. 3*A*, red data points). H3K9me3 enrichment change at three representative transposons (*gypsy*, *diver*, and *gtwin*) was further validated through quantitative PCR (qPCR). The ribosomal protein gene *rp49*, a transcriptionally active locus, was a negative control for H3K9me3 enrichment analysis. The H3K9me3 mark exhibited a minimum 50-fold enrichment at the three transposons compared to *rp49* (Fig. 3*B*). H3K9me3 levels at *gypsy*, *diver*, and *gtwin* decreased by approximately fivefold in the absence of Modulo (Fig. 3*B*, compare gray and pink columns). A comparable decrease in H3K9me3 levels at these three transposons was observed in *piwi*^*2/dNLS*^ ovaries (Fig. 3*B*, compare green and purple columns). These findings indicate that Piwi and Mod are equally critical for the deposition of H3K9me3 at the three transposons examined. Furthermore, genomic analysis indicated that the absence of Mod function disrupts H3K9me3 deposition at the majority of transposon insertions when compared to the heterozygous control *mod*^*07570*^/+ (Fig. 3*C*, heat map, compare *mod*^*07570/L8*^ with *mod*^*07570*^/+). H3K9me3 levels at genes were low compared to transposons and remained similar regardless of Mod’s presence (Fig. 3*D*, meta plots compare blue and green profiles). An analysis of H3K9me3 enrichment at a specific genomic location on chromosome 2L corroborated our findings (Fig. 3*E*). Reads from H3K9me3 ChIP that uniquely mapped to the genome were used to identify peaks with MACS2 (43). The quantity of unique mapping reads, and the associated peaks significantly decline in *mod*^*07570/L8*^ relative to *mod*^*07570*^/+ (Fig. 3*E*, shaded boxes). Finally, we tested if Mod’s role in H3K9me3 enrichment at transposons is direct or indirect. To this end, we tested if the Mod protein is localized to transposon genomic sites using ChIP-qPCR. Isotype IgG was used as a control. Mod enrichment at *diver*, *gtwin*, and *gypsy* transposons was calculated as fraction input and compared between control and experimental samples. Mod was enriched at these transposons ∼3 to 5 fold (*p-value* < 0.01) compared to the isotype IgG control (Fig. 3*F*, compare gray and pink columns). Based on these results, we conclude that Mod is recruited to transposon genomic sites and is essential for H3K9me3 enrichment at transposons.

**Figure 4:**
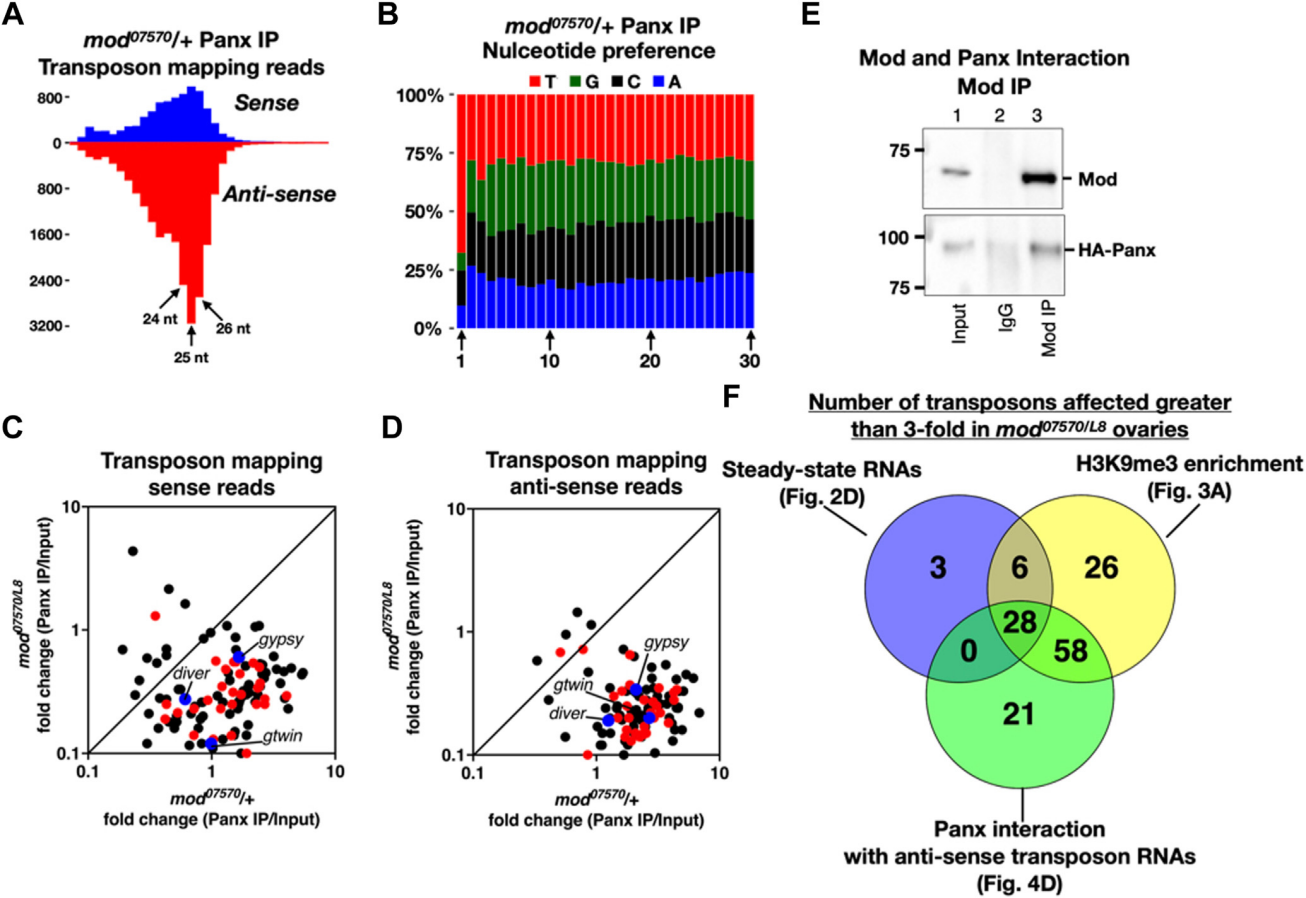
**Mod is needed for Panx’s ability to target transposons.***A*, a column plot showing the number of transposon-mapping sense (*blue*) and anti-sense (*red*) small RNAs bound to Panx in the heterozygous control *mod*^*07570*^*/+*. *B*, relative enrichment of nucleotides at various positions along the length of Panx-bound small RNAs is shown. Nucleotide positions are shown at the *bottom*. *C* and *D*, scatter plots showing fold-change (Panx IP/Input) in transposon mapping sense (*C*) and anti-sense (*D*) reads in *mod*^*07570*^/+ and *mod*^*07570/L8*^ ovaries. Data points in red indicate transposons activated greater than 3-fold in Fig. 2*D*. *gypsy*, *diver*, and *gtwin* transposons are marked in *blue*. *E*, immunoblot result showing the interaction between Mod and Panx in ovary lysates. Panx was detected using an antibody against the HA epitope tag. *F*, a Venn diagram comparing results from genomics experiments performed in this work. The number of transposons affected greater than 3-fold are compared. Please note that the size of the circles does not correspond to the size of the data sets they represent.

**Figure 6:**
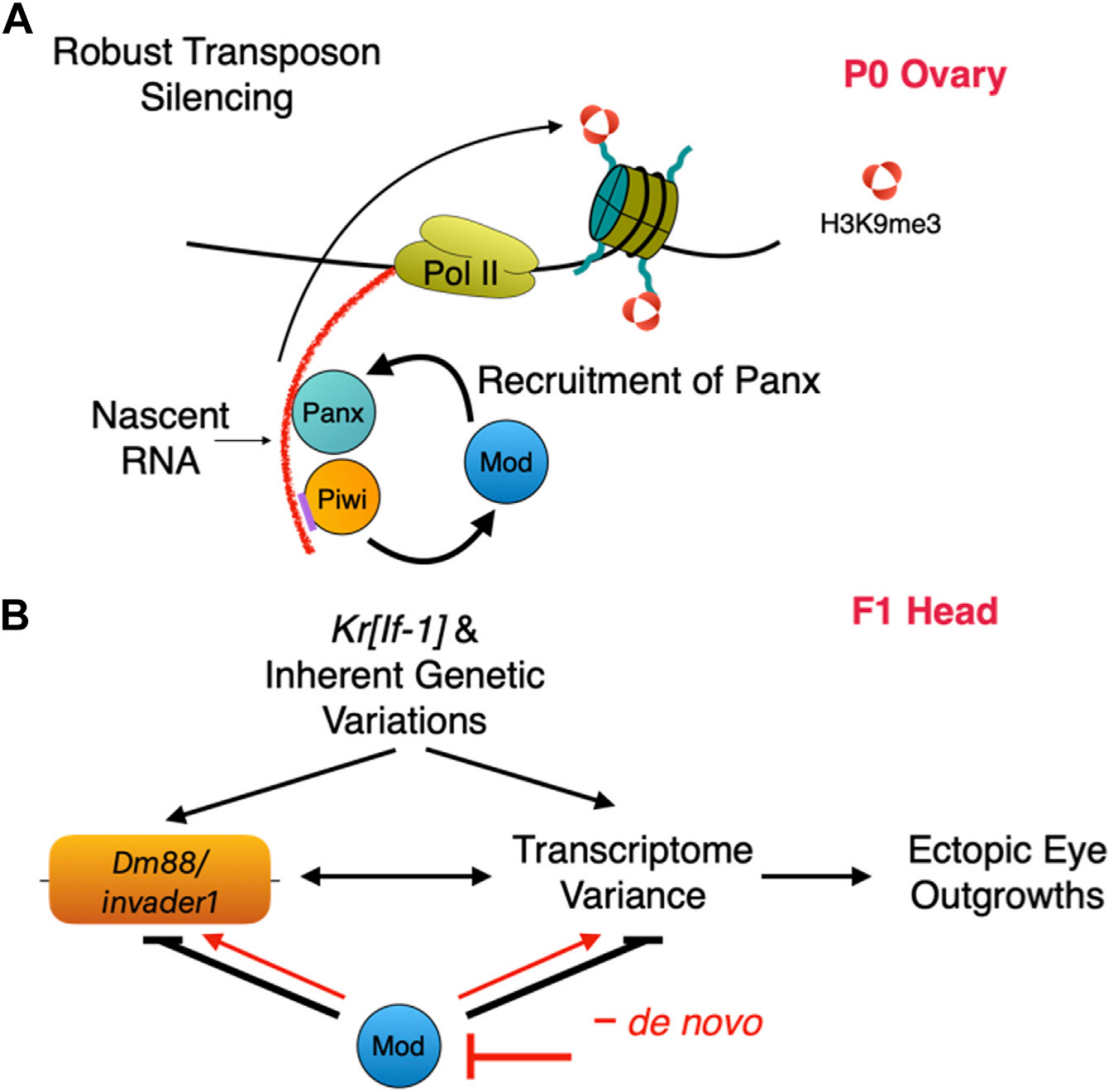
**A model for Piwi and Modulo's role in transposon silencing and developmental robustness.***A*, a model illustrating the functional relationship among Piwi, Mod, and Panx leading to H3K9me3 enrichment at transposon genomic sites. *B*, an illustration showing how Mod prevents ectopic eye outgrowths by negating the effect of *Kr[If-1]* and inherent genetic variations on transposon silencing and transcriptome variance. *Red* lines show the alternate model where the lack of Mod function induces *de novo* variations.

We then inquired whether Modulo's role in transposon silencing involves altering the piRNA pathway function. Recruitment of the Piwi-associated component Panoramix (Panx) to target transposon RNA results in robust heterochromatin formation and reporter silencing (14, 15). Specifically, using sequencing of Panx-associated small RNAs, Panx has been shown to interact with target transposon RNAs (15). We thus tested if Mod regulates Panx’s association with target transposon RNAs. To this end, we isolated and sequenced Panx-associated small RNAs in the presence and absence of Modulo. As previously reported, the Panx-associated transposon-mapping small RNAs are predominantly 25 nt in length (length of piRNAs) and anti-sense (Fig. 4*A*, compare and blue and red columns. Also see Fig. S3*A*). They also preferentially start with Uracil at the first position (Fig. 4*B*). Next, a comparison in *mod*^*07570*^/+ and *mod*^*07570/L8*^ ovaries shows that Panx binding to both sense and anti-sense transposon mapping small RNAs decreases drastically in the absence of Modulo (Fig. 4, *C* and *D*). Sense and anti-sense read mapping to 79 and 110 transposons was reduced by more than 3-fold in the absence of Modulo (Fig. 4, *C* and *D*). This result shows that Modulo is essential for Panx’s ability to target transposons. Furthermore, a co-immunoprecipitation experiment showed that Modulo and Panx interact (Figs. 4*E* and S3*B*, compare lanes 2 and 3 in Mod and Panx panels). However, the reciprocal co-immunoprecipitation experiment did not replicate Mod and Panx interaction (Fig. S3*C*, compare lanes 2 and 3 in anti-Mod and anti-Panx panels). It could be because of several reasons, including (1) transient interaction between Panx and Mod, (2) differences in the protein expression levels of Panx and Mod, and (3) HA antibody disrupting Panx and Mod interaction. In conclusion, these results show that Modulo acts upstream of Panx and helps Panx target transposons (Fig. 6*A*). A comparison of various genomics results from this study indicates that Mod is needed for epigenetic silencing of a vast majority of transposons (Fig. 4*F*). Panx’s association with anti-transposon small RNAs and H3K9me3 enrichment at 86 transposons depend on Mod. Intriguingly, only 28 out of these 86 transposons exhibit increased steady-state mRNA levels; the exact reason remains to be identified.

**Figure 5:**
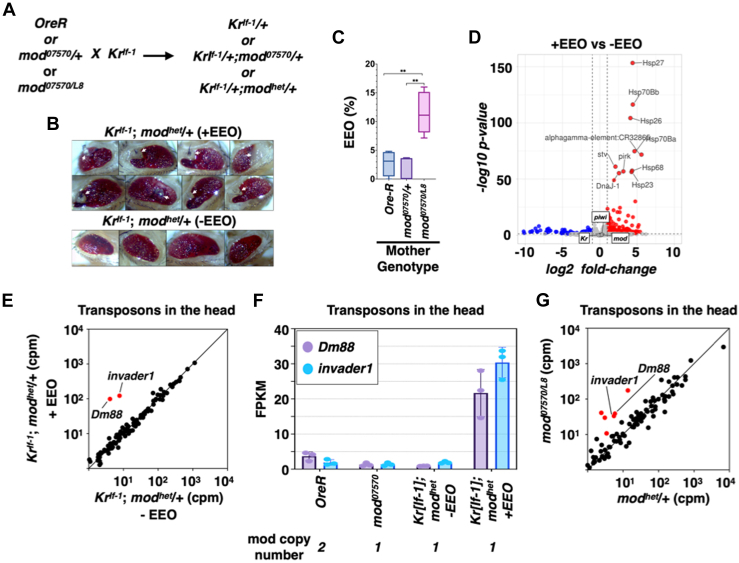
**Modulo is essential for developmental robustness.***A*, crossing scheme to test if lack of Mod function in the ovary leads to ectopic outgrowths (EEOs) in the offspring. *B*, bright-field microscope images of fly eyes with and without EEOs. The *white* arrows mark EEOs. *C*, a box plot showing the quantitation of EEOs observed in Fig. 5*A*. The mother's genotype is shown at the *bottom*. *N* = 259 (*Ore-R*), 119 (*mod*^*07570*^*/+*), and 114 (*mod*^*07570*^*/mod*^*L8*^) ∗∗ *p-value* less than 0.05. The experiment had at least 3 biological replicates for each genotype and the *p-value* was calculated using an unpaired *t* test. *D*, a volcano plot showing the differential expression of genes in the presence and the absence of EEO. The top 10 differentially expressed genes are marked. The experiment had three biological replicates. *E*, a scatter plot showing the steady-state levels of transposon mRNAs in the presence and absence of EEO. Transposons differentially expressed greater than 5-fold are marked in *red*. Reads were normalized as counts per million (cpm) reads that mapped to the *dm3* genome. The average of 3 biological replicates is shown. *F*, a column plot showing the mRNA fragments per kilobase of transcript per million mapped reads (FPKM) of *Dm88* and *invader1* transposons in various genotypes shown at the *bottom*. *mod* copy number in respective genotypes is also shown. Individual data points represent three independent biological replicates. *G*, same as in *E* except that heads from *mod*^*07570*^ and *mod*^*07570/L8*^ were compared. Transposons differentially expressed greater than 5-fold are marked in *red*. The average of three biological replicates is shown. EEO, ectopic eye outgrowths.

Finally, we tested if Mod, like Piwi, is needed for developmental robustness. To this end, *mod*^*07570/L8*^ females were mated with *Kr*^*If-1*^males, and the offspring were evaluated for ectopic eye outgrowth (EEO) phenotypes. The reference flies were *Oregon-R* and *mod*^*07570*^*/+* (Fig. 5*A*). The absence of Mod in the mother resulted in EEO in the offspring (Fig. 5*B*, white arrows). *mod*^*07570/L8*^ mothers generated four times more EEO in their offspring than either *Oregon-R* or *mod*^*07570*^*/+* mothers (Fig. 5*C*). Based on these findings, we conclude that Mod is essential for developmental robustness. Comparison of head transcriptomes showed that EEOs are not due to a variegated effect on the expression of *Kr*, *piwi*, or *mod* genes, as we did not see any change in the expression levels of these genes in the presence of EEOs (Fig. 5*D*). However, we noticed a significant upregulation in the expression of several heat shock proteins, with 7 of the top 10 differentially expressed genes comprised of these stress response pathway proteins. The significance of upregulation of stress response pathway genes is yet to be determined. In addition to the transcriptome variation, the flies with EEOs were associated with the activation of 2 specific transposons—*Dm88* and *invader1*, with both being activated more than ∼15-fold (Fig. 5*E*, red data points) in the presence of EEOs. *Dm88* and *invader1* were activated only in the presence of the EEO; flies without the EEO had similar levels of these transposons as in control flies, *OreR,* and *mod*^*07570*^ (Fig. 5*E*). Thus, the activation of *Dm88* and *invader1* is strongly associated with the incidence of EEO. The lack of activation of these transposons in flies without EEOs suggests that these transposons are activated due to inherent genetic variations or *de novo* genetic and epigenetic variations in flies with the EEOs. Interestingly, both *Dm88* and *invader1* are repressed by Mod. Loss of both copies of the *mod* gene activated *Dm88* and *invader1* ∼6-fold compared to *mod*^*07570*^/+ heads (Fig. 5*G*). Based on these results, we conclude that EEO in the absence of Mod is in part due to the lack of robust silencing of *Dm88* and *invader1*. Our data also shows that *mod* is halposufficient to silence *Dm88* and *invader1* in the heads (Fig. 5*F*), but one copy of *mod* is insufficient to prevent the activation of these two transposons in the progeny with EEO. Thus, the Mod's function in the mother dictates its ability to promote robust transposon silencing and developmental robustness in the next generation.

## Discussion

In this study, we identified a novel function for the Mod protein in transposon silencing and promoting developmental robustness.

The molecular mechanism that governs Piwi-mediated transcriptional silencing of transposons is gradually being revealed *via* elegant research from various laboratories. Multiple proteins–Maelstrom, Asterix, Panx (as part of the SFiNX/PICTS complex), and Su(var)2-10–promote the nuclear function of Piwi-mediated transcriptional silencing of transposons (10, 11, 12, 13, 14, 15, 16, 17, 18, 19, 20, 21). Our study adds Mod to this list of nuclear factors that promote Piwi function in transposon silencing. It shows that Mod functions downstream of Piwi localization to the nucleus and upstream of Panx recruitment to transposons(Fig. 6*A*). Panx’s ability to target transposon RNAs depends on Mod. We propose that Piwi interaction with Mod (Fig. 1, *C* and *D*) is needed for the latter’s recruitment to transposon genomic sites. However, the alternate mechanism that Mod recruitment to transposons is independent of Piwi and Piwi-Mod interaction has a non-transposon silencing function cannot be ruled out yet. Further structure-function studies and characterization of interaction interface mutants are needed to answer this question. We show Mod localizes to transposon genomic sites (Fig. 3*F*); the exact mechanism remains to be studied. Elegant RNA and DNA tethering experiments developed by the Hannon and Brenneckke labs should help answer this question (14, 15). Unraveling the mechanism by which Mod helps Panx target transposon RNAs (Fig. 4, *C* and *D*) is also essential. It is also unclear if Mod interacts with Panx independently or as a part of the SFiNX/PICTS complex (18). It is intriguing to notice that of the 86 transposons with greatly diminished H3K9me3 levels and Panx-associated small RNAs, only 28 exhibited a greater than 3-fold increase in steady-state mRNA levels. We believe that this discrepancy could be because of two reasons—(1) a robust post-transcriptional silencing by Aub and Ago3 and/or (2) a diminished recruitment of RNA pol II to transposon sites. Furthermore, recent work has also shown that Mod regulates centromere assembly at the nucleolus (44, 45), which may have implications for properly silencing centromeric repeats and transposons. It will be interesting to see if this Mod function in centromere positioning regulates transposon silencing. *Drosophila* Modulo is the homolog of mammalian Nucleolin which, like Modulo, is associated with chromatin (46). Nucleolin has been shown to act as a histone chaperone and to increase the remodeling efficiency of ATP-dependent chromatin remodeling (47). It has also been shown to act like the Facilitates Chromatin Transcription (FACT) complex and helps pass RNA polymerase II through nucleosomes. Nucleolin has also been shown to be essential for nucleosome disruption that precedes double-strand break repair (48). Based on Nucleolin’s role in chromatin remodeling, it will be interesting to test if Nucleolin, in mammals, is also needed for transposon silencing.

Our study also shows that Mod, like Piwi, is essential for developmental robustness. Flies with the EEOs had two distinct signatures—(1) transcriptome variation with activated stress response genes (Fig. 5*D*) and (2) higher steady-state mRNA levels of *Dm88* and *invader1* (Fig. 5*E*). It remains to be tested if these changes are restricted to the EEO or occur in the flies before developing EEOs. Nevertheless, our data shows that flies with EEOs were poised for transposon activation and transcriptome variation. Recent elegant work has demonstrated that genes and transposons can regulate each other’s expression (5, 49). It remains to be seen if transcriptome variance causes *Dm88* and *invader1* activation or *vice versa*. Important to note is that the level of *Dm88* and *invader1* activation in the presence of EEO is much higher than what we observed in the ovaries; perhaps due to the absence of essential piRNA pathway proteins. We also show that a few transposons in the heads are activated greater than 5-fold in the absence of Mod. The mechanism by which Mod silences transposons in the heads in the absence of piRNA pathway components is unclear and needs to be defined. How are flies with EEOs poised for transposon activation and transcriptome variance? We propose two mechanisms are at play here (Fig. 6*B*). First, *Kr[If-1]* and inherent genetic variations can induce transposons and transcriptome variation. The robust maternal function of Mod prevents the effect of inherent genetic variations and promotes developmental robustness. An alternate mechanism is that a lack of robust Mod function in the mother and early embryos can cause *de novo* genetic and epigenetic variations, leading to compromised transposon silencing and transcriptome fidelity (Fig. 6*B*, red lines). Transposon insertion-based *de novo* genetic mutations have been shown to cause phenotype variations (4). Thus, checking if the compromised Mod function causes transposon movement in the genome will be necessary. Using a genetic screen for maternal enhancers of *Kr[If*-*1]*, Ruden lab has shown that epigenetic regulation by the *trithorax* (TrxG) group of proteins can promote developmental robustness (32). It will be interesting to see if Mod regulates TrxG function and if the lack of the same leads to *de novo* epigenetic variations. Thus, our work lays the foundation for future research that unravels the connections among Mod function in transposon silencing, predisposition to transposon activation in the offspring, transcriptome variation, stress response pathway function, and developmental robustness.

## Experimental procedures

### Fly stocks and maintenance

All *Drosophila* stocks were maintained at 25 °C. The fly stocks used in this study were as follows: *Kr*^*If-1*^ (#4194), *Oregon-R* (#25211), *mod*^*07570*^/*TM3* (#11795), and *mod*^*L8*^/*TM3* (#383432). All fly stocks were obtained from the Bloomington stock center (stock numbers are indicated in parentheses).

### Generation of UAS-HA-Panx flies

The Panx coding sequence (with the HA-epitope tag after the first amino acid) was amplified from ovary cDNA using the primers 5′-GGGGACAAGTTTGTACAAGAAAGCAGGCTTCACCATGGCCATGTACCCATACGACGTCCCAGACTACGCTGAAGCTCCGATGAAGCT-3′ and 5′-GGGGACCACTTTGTACAAGAAAGCTGGGTCCTATGGCTGTCGACCCTTTATTATTG-3′ and cloned into pDONR221 and then into pPFW plasmid. BestGene Inc (Chino Hills, California) injected pPFW plasmid into *w*^*1118*^ embryos to create transgenic UAS-HA-Panx lines.

### Antibodies

All antibodies used in this work are shown in Table S1. In addition, a rabbit polyclonal antibody against the Mod peptide SHRSNKHQEESRKR was generated using services rendered by GenScript. Mod antibody was validated using immunoblot analysis and confocal microscopy. Please see Figure 1, *B* and *C*.

### Immunoprecipitation (IP)

Fifty pairs of ovaries were utilized for each IP experiment. The ovaries were dissected in ice-cold 1X phosphate-buffered saline (PBS). PBS was substituted with 50 μl OLB (30 mM HEPES at pH 8.0, 150 mM potassium acetate, 5 mM magnesium acetate, 5 mM DTT, 0.1% NP-40, and 5% glycerol) and manually homogenized in a 1.5 ml microcentrifuge tube. The volume was adjusted to 500 μl using OLB and centrifuged at 1100*g* for 2 min. The supernatant was retained as a cytoplasmic fraction, while the pellet was resuspended in 500 μl of NLB (50 mM Tris-HCl at pH 7.5, 20 mM NaCl, 1 mM MgCl_2_, and 1% NP-40) and sonicated three times for 15 s each. Benzonase (Millipore Sigma, #E1014) was added to a concentration of 1:1000, and the crude nuclear lysate was incubated at 4 °C for 1 h on a tube rotator. The crude nuclear lysate was then centrifuged at 16,000*g* for 10 min, and the supernatant was retained as nuclear lysate for IP. In each IP experiment, the nuclear lysate was divided into two equal aliquots; one was incubated with an antibody targeting the protein of interest, while the other was incubated with control isotype IgG. Incubation was conducted overnight at 4 °C on a tube rotator. To the lysate-antibody mixture, 25 μl of Protein A/G Magnetic Beads (ThermoFisher, #88802) were added and incubated for 3 h at 4 °C on a tube rotator. The beads were washed three times with OLB and subsequently processed for immunoblotting. All buffers were supplemented with protease inhibitors (ThermoFisher, # A32965).

### Immunofluorescence

Immunofluorescence experiments were performed as previously described (50). In brief, ovaries were dissected and fixed in 4% formaldehyde in PBS for 20 min at room temperature, after which they were washed three times with Phosphate-buffered saline (PBS) and three times with PBS+ 0.2% Triton X-100 (PBT). Ovaries were dissociated by gently pipetting 2 to 3 times using a cut pipette tip. Then, permeabilization and blocking were carried out by first incubating the samples with PBT for 20 min, followed by incubation with PBT+1% BSA for 1 h at room temperature. Primary antibodies were used at the following dilutions: Piwi (1:500), Fibrillarin (1:1000), and Mod (1:500). Secondary antibodies (Alexa Fluor-conjugated 488, 566, and 633) were purchased from Invitrogen and were used at a 1:500 dilution. Ovaries were incubated with primary antibody overnight at 4 °C in PBT + 1% BSA, washed three times for 1 h each with PBT, and incubated with secondary antibody overnight at 4 °C, followed by three washes each for 15 min using PBT. Ovaries were mounted with a DAPI mounting solution. Confocal images were taken using a Zeiss LSM 880 NLO microscope and Plan-Apochromat 63x/1.40 Oil DIC objective with identical control and test fly lines settings. Images were analyzed using ImageJ.

### mRNA-seq

Total RNA was extracted from ovaries or heads using TRIzol (Invitrogen) following the manufacturer’s instructions. RNA integrity was verified by running samples on an Agilent 2100 bioanalyzer with an RNA integration number ≥ 8. Then, 100 to 200 ng of RNA was used for mRNA sequencing (mRNA-seq) using the Illumina PE150 strategy.

### Sequencing of Piwi-bound small RNAs

Whole ovary lysate was prepared from 50 pairs of ovaries as described elsewhere (33). Immunoprecipitation with the Piwi antibody was performed, and to extract small RNAs bound to Piwi, the beads, after the washing step, were treated with TRIzol, and the total RNA was extracted and submitted for sequencing. Prior to library preparation and sequencing, the RNA sample was supplemented with 1 μl of 10 μM 2S Block oligo (5′- TAC AAC CCT CAA CCA TAT GTA GTC CAA GCA/3SpC3/-3′) to inhibit amplification of ribosomal RNA selectively. RNA sequencing was performed using the Illumina SE50 strategy.

### ChIP-seq

Approximately 50 μl ovaries were crosslinked with 1% formaldehyde in PBS for 10 min at room temperature, quenched with 150 mM glycine for 5 min, and washed three times, 5 min each, in PBT. Lysis was performed in 500 μl of lysis buffer (10 mM Tris-HCl at pH 8, 100 mM NaCl, 1 mM EDTA, 0.1% sodium deoxycholate, 0.5% N lauroylsarcosine, 1 mM DTT and 1x Halt Protease Inhibitor from Thermo). Ovaries were homogenized on a 1 ml Wheaton homogenizer with 5 loose and 5 tight strokes and kept on ice for 30 min. Lysates were prepared as 100 μl aliquots and sonicated on a Bioruptor (Diagenode) for 10 cycles at high strength with 30 s ON and 45 s OFF pulses. The lysates were then pooled, and the volume was adjusted to 1 ml with lysis buffer, followed by centrifugation at 12,000 rpm for 10 min at 4 °C. The supernatants containing the soluble chromatin were transferred to new tubes, and 100 μl was saved as input control. Immunoprecipitation was carried out by adding 2 μg of anti-H3K9me3 (Abcam, ab8898) to the respective supernatants and incubating overnight on a rotating wheel at 4 °C. Protein A/G Magnetic Beads (ThermoFisher, #88802) were added to the supernatant-antibody mix, and the samples were incubated for an additional 3 h at 4 °C. The ChIP samples were washed five times: once with low-salt buffer (20 mM Tris-HCl at pH 8, 150 mM NaCl, 2 mM EDTA, 0.1% SDS, and 0.1% Triton-X 100), twice with high-salt buffer (20 mM Tris-HCl at pH 8, 500 mM NaCl, 2 mM EDTA, 0.1% SDS, and 0.1% Triton-X 100), once with LiCl buffer (10 mM Tris-HCl at pH 8, 1 mM EDTA, 1% NP40, 1% deoxycholate and 250 mM LiCl) and once with TE buffer (10 mM Tris-HCl at pH 8 and 1 mM EDTA). Then, the beads were resuspended in 100 μl of elution buffer (50 mM Tris-HCl at pH 8, 10 mM EDTA, 1% SDS, and 300 mM NaCl). The input samples were thawed, mixed with an equal volume of elution buffer, and processed in parallel with the ChIP samples. Crosslinking was reversed by incubating the beads and inputs overnight at 65 °C. The samples were sequentially treated with 2 μl of RNAseA + T1 (Thermo, EN0551) for 30 min at 37 °C, and 5 μl of Proteinase K (Thermo, EO0491) for 2 h at 37 °C, and the immunoprecipitated DNA was then extracted using a PCR purification kit (Qiagen). ChIP was repeated thrice with three independent biological replicates. One replicate was sequenced using the Illumina SE150 strategy, and all three biological replicates were used for qPCR.

### ChIP qPCR

ChIP qPCR was performed using primers shown in Table S2. The relative enrichment of H3K9me3 was calculated using the formula 2∧-(Ct^ChIP^-Ct^Input^). The ribosomal protein gene *rp49* was used as a negative control. All qPCR reactions were performed in 10 μl using SyberGreen Supermix (Biorad, 1725271) and the CFX384 real-time PCR detection system (Bio-Rad).

### Bioinformatics

#### RNA-seq analysis

Reads were directly mapped to transposon consensus sequences and gene transcripts using *Bowtie 2* (51) and then quantified using *eXpress* (52). Estimated counts were normalized as counts per million (cpm). All mRNA sequencing experiments performed in this study had three biological replicates. For Fig. 5*D*, reads were mapped to the *dm3* genome using STAR (53) and quantified using htseq-count (54). Differential expression analysis was performed using DESeq2 (55). Analysis was performed on the Galaxy platform (56). The volcano plot in Fig. 5*D* was generated using VolcaNoseR (57). All other plots were generated using GraphPad Prism. Sequence read archives files SRR10541133 and SRR10541133 (42) were used as input files for *piwi*^*2/dNLS*^ in Fig. 2*F*.

#### ChIP-seq data analysis

In Fig. 3*A*, ChIP-seq analysis was performed using piPipes (58). As part of the piPipes workflow, ChIP and input reads were mapped to transposon consensus sequences and gene transcripts using *Bowtie 2* (51) and then quantified using *eXpress* (52). Estimated counts were normalized as counts per million (cpm). Fold enrichment was calculated using the formula ChIP^cpm^/Input^cpm^ and plotted. Figure 3, *C*–*E* were generated using deepTools (59). Sequencing reads were first mapped to the *dm3* genome using *Bowtie* (60). *Bowtie* was executed with default parameters to identify all genome mapping reads, and the ‘*-m 1*’ option was employed to detect reads that mapped only once in the genome (unique reads). The sorted and indexed BAM files were then processed using *bamCompare* (*--normalizeUsing CPM*), *computeMatrix* (using *scale-regions*, -*-beforeRegionStartLength 1000*, *--regionBodyLength* 1000 -*-afterRegionStartLength 1000*, *--skipZeros* options), and *plotHeatmap* tools. Genomic locations (BED files) of the transposons and genes were downloaded using the UCSC Table Browser. Integrative Genomics Viewer (61) was used to generate the genome browser views in Fig. 3*E*.

#### Small RNA seq data analysis

The 3′-adapter 5′-AGATCGGAAGAGCACACGTCT-3′ was trimmed from the sequenced reads using Cutadapt version 1.15. Trimmed reads less than 18 nucleotides and reads without the adapter were discarded. Trimmed reads were then analyzed using the piPipes small RNA pipeline (58). In Fig. 2*C*, reads were mapped to genes and transposon consensus sequences using *Bowtie 2* and quantified by eXpress, as mentioned earlier. Estimated counts were normalized as cpm and plotted. In Fig. S2*B*, sense and anti-sense reads mapping to transposons were normalized to genome mapping reads. Size distribution was analyzed using *TBr2_basicanalyses.pl* (62) and plotted. The annotation shown in Fig. S2*C* was done using piPipes. In Figure 4, *C* and *D*, small RNAs associated with Panx were normalized as fraction input and plotted. Size distribution in Fig. 4*A* and the first nucleotide signature in Fig. 4*B* were identified using piPipes.

## Data availability

All data needed to evaluate the conclusions in the paper are present in the paper and/or the Supplementary Materials. Sequencing data from this study can be accessed using Gene Expression Omnibus (GEO) accession number GSE240478.

## Supporting information

This article contains supporting information.

## Conflict of interests

The authors declare that they have no conflicts of interest with the contents of this article.

## References

[1] Chuong E.B., Elde N.C., Feschotte C. (2017). Regulatory activities of transposable elements: from conflicts to benefits. Nat. Rev. Genet..

[2] Yang N., Srivastav S.P., Rahman R., Ma Q., Dayama G., Li S. (2022). Transposable element landscapes in aging Drosophila. PLoS Genet..

[3] Pezic D., Manakov S.A., Sachidanandam R., Aravin A.A. (2014). piRNA pathway targets active LINE1 elements to establish the repressive H3K9me3 mark in germ cells. Genes Dev..

[4] Specchia V., Piacentini L., Tritto P., Fanti L., D'Alessandro R., Palumbo G. (2010). Hsp90 prevents phenotypic variation by suppressing the mutagenic activity of transposons. Nature.

[5] Hummel B., Hansen E.C., Yoveva A., Aprile-Garcia F., Hussong R., Sawarkar R. (2017). The evolutionary capacitor HSP90 buffers the regulatory effects of mammalian endogenous retroviruses. Nat. Struct. Mol. Biol..

[6] Ghildiyal M., Seitz H., Horwich M.D., Li C., Du T., Lee S. (2008). Endogenous siRNAs derived from transposons and mRNAs in Drosophila somatic cells. Science.

[7] Ozata D.M., Gainetdinov I., Zoch A., O'Carroll D., Zamore P.D. (2019). PIWI-interacting RNAs: small RNAs with big functions. Nat. Rev. Genet..

[8] Czech B., Munafo M., Ciabrelli F., Eastwood E.L., Fabry M.H., Kneuss E. (2018). piRNA-Guided genome defense: from biogenesis to silencing. Annu. Rev. Genet..

[9] Wang C., Lin H. (2021). Roles of piRNAs in transposon and pseudogene regulation of germline mRNAs and lncRNAs. Genome Biol..

[10] Donertas D., Sienski G., Brennecke J. (2013). Drosophila Gtsf1 is an essential component of the Piwi-mediated transcriptional silencing complex. Genes Dev..

[11] Ohtani H., Iwasaki Y.W., Shibuya A., Siomi H., Siomi M.C., Saito K. (2013). DmGTSF1 is necessary for Piwi-piRISC-mediated transcriptional transposon silencing in the Drosophila ovary. Genes Dev..

[12] Muerdter F., Guzzardo P.M., Gillis J., Luo Y., Yu Y., Chen C. (2013). A genome-wide RNAi screen draws a genetic framework for transposon control and primary piRNA biogenesis in Drosophila. Mol. Cell.

[13] Sienski G., Donertas D., Brennecke J. (2012). Transcriptional silencing of transposons by Piwi and maelstrom and its impact on chromatin state and gene expression. Cell.

[14] Yu Y., Gu J., Jin Y., Luo Y., Preall J.B., Ma J. (2015). Panoramix enforces piRNA-dependent cotranscriptional silencing. Science.

[15] Sienski G., Batki J., Senti K.A., Donertas D., Tirian L., Meixner K. (2015). Silencio/CG9754 connects the Piwi-piRNA complex to the cellular heterochromatin machinery. Genes Dev..

[16] Murano K., Iwasaki Y.W., Ishizu H., Mashiko A., Shibuya A., Kondo S. (2019). Nuclear RNA export factor variant initiates piRNA-guided co-transcriptional silencing. EMBO J..

[17] Fabry M.H., Ciabrelli F., Munafo M., Eastwood E.L., Kneuss E., Falciatori I. (2019). piRNA-guided co-transcriptional silencing coopts nuclear export factors. Elife.

[18] Batki J., Schnabl J., Wang J., Handler D., Andreev V.I., Stieger C.E. (2019). The nascent RNA binding complex SFiNX licenses piRNA-guided heterochromatin formation. Nat. Struct. Mol. Biol..

[19] Onishi R., Sato K., Murano K., Negishi L., Siomi H., Siomi M.C. (2020). Piwi suppresses transcription of Brahma-dependent transposons via Maelstrom in ovarian somatic cells. Sci. Adv..

[20] Eastwood E.L., Jara K.A., Bornelov S., Munafo M., Frantzis V., Kneuss E. (2021). Dimerisation of the PICTS complex via LC8/Cut-up drives co-transcriptional transposon silencing in Drosophila. Elife.

[21] Schnabl J., Wang J., Hohmann U., Gehre M., Batki J., Andreev V.I. (2021). Molecular principles of Piwi-mediated cotranscriptional silencing through the dimeric SFiNX complex. Genes Dev..

[22] Waddington C.H. (1959). Canalization of development and genetic assimilation of acquired characters. Nature.

[23] Zabinsky R.A., Mason G.A., Queitsch C., Jarosz D.F. (2019). It's not magic - Hsp90 and its effects on genetic and epigenetic variation. Semin. Cell Dev. Biol..

[24] Felix M.A., Wagner A. (2008). Robustness and evolution: concepts, insights and challenges from a developmental model system. Heredity (Edinb).

[25] Bergman A., Siegal M.L. (2003). Evolutionary capacitance as a general feature of complex gene networks. Nature.

[26] Hornstein E., Shomron N. (2006). Canalization of development by microRNAs. Nat. Genet..

[27] Salathia N., Queitsch C. (2007). Molecular mechanisms of canalization: Hsp90 and beyond. J. Biosci..

[28] Rutherford S.L., Lindquist S. (1998). Hsp90 as a capacitor for morphological evolution. Nature.

[29] Jarosz D.F., Lindquist S. (2010). Hsp90 and environmental stress transform the adaptive value of natural genetic variation. Science.

[30] Queitsch C., Sangster T.A., Lindquist S. (2002). Hsp90 as a capacitor of phenotypic variation. Nature.

[31] Rohner N., Jarosz D.F., Kowalko J.E., Yoshizawa M., Jeffery W.R., Borowsky R.L. (2013). Cryptic variation in morphological evolution: HSP90 as a capacitor for loss of eyes in cavefish. Science.

[32] Sollars V., Lu X., Xiao L., Wang X., Garfinkel M.D., Ruden D.M. (2003). Evidence for an epigenetic mechanism by which Hsp90 acts as a capacitor for morphological evolution. Nat. Genet..

[33] Gangaraju V.K., Yin H., Weiner M.M., Wang J., Huang X.A., Lin H. (2011). Drosophila Piwi functions in Hsp90-mediated suppression of phenotypic variation. Nat. Genet..

[34] Garzino V., Pereira A., Laurenti P., Graba Y., Levis R.W., Le Parco Y. (1992). Cell lineage-specific expression of modulo, a dose-dependent modifier of variegation in Drosophila. EMBO J..

[35] Graba Y., Laurenti P., Perrin L., Aragnol D., Pradel J. (1994). The modifier of variegation modulo gene acts downstream of dorsoventral and HOM-C genes and is required for morphogenesis in Drosophila. Dev. Biol..

[36] Karam J.A., Parikh R.Y., Nayak D., Rosenkranz D., Gangaraju V.K. (2017). Co-chaperone Hsp70/Hsp90-organizing protein (Hop) is required for transposon silencing and Piwi-interacting RNA (piRNA) biogenesis. J. Biol. Chem..

[37] Parikh R.Y., Lin H., Gangaraju V.K. (2018). A critical role for nucleoporin 358 (Nup358) in transposon silencing and piRNA biogenesis in Drosophila. J. Biol. Chem..

[38] Perrin L., Romby P., Laurenti P., Berenger H., Kallenbach S., Bourbon H.M. (1999). The Drosophila modifier of variegation modulo gene product binds specific RNA sequences at the nucleolus and interacts with DNA and chromatin in a phosphorylation-dependent manner. J. Biol. Chem..

[39] Spradling A.C., Stern D., Beaton A., Rhem E.J., Laverty T., Mozden N. (1999). The Berkeley Drosophila Genome project gene disruption project: single P-element insertions mutating 25% of vital Drosophila genes. Genetics.

[40] Perrin L., Demakova O., Fanti L., Kallenbach S., Saingery S., Mal'ceva N.I. (1998). Dynamics of the sub-nuclear distribution of Modulo and the regulation of position-effect variegation by nucleolus in Drosophila. J. Cell Sci..

[41] Falahati H., Wieschaus E. (2017). Independent active and thermodynamic processes govern the nucleolus assembly in vivo. Proc. Natl. Acad. Sci. U. S. A..

[42] Zhang G., Yu T., Parhad S.S., Ho S., Weng Z., Theurkauf W.E. (2021). piRNA-independent transposon silencing by the Drosophila THO complex. Dev. Cell.

[43] Zhang Y., Liu T., Meyer C.A., Eeckhoute J., Johnson D.S., Bernstein B.E. (2008). Model-based analysis of ChIP-seq (MACS). Genome Biol..

[44] Padeken J., Mendiburo M.J., Chlamydas S., Schwarz H.J., Kremmer E., Heun P. (2013). The nucleoplasmin homolog NLP mediates centromere clustering and anchoring to the nucleolus. Mol. Cell.

[45] Chen C.C., Greene E., Bowers S.R., Mellone B.G. (2012). A role for the CAL1-partner Modulo in centromere integrity and accurate chromosome segregation in Drosophila. PLoS One.

[46] Mongelard F., Bouvet P. (2007). Nucleolin: a multiFACeTed protein. Trends Cell Biol..

[47] Angelov D., Bondarenko V.A., Almagro S., Menoni H., Mongelard F., Hans F. (2006). Nucleolin is a histone chaperone with FACT-like activity and assists remodeling of nucleosomes. EMBO J..

[48] Goldstein M., Derheimer F.A., Tait-Mulder J., Kastan M.B. (2013). Nucleolin mediates nucleosome disruption critical for DNA double-strand break repair. Proc. Natl. Acad. Sci. U. S. A..

[49] Treiber C.D., Waddell S. (2020). Transposon expression in the Drosophila brain is driven by neighboring genes and diversifies the neural transcriptome. Genome Res..

[50] Preall J.B., Czech B., Guzzardo P.M., Muerdter F., Hannon G.J. (2012). Shutdown is a component of the Drosophila piRNA biogenesis machinery. RNA.

[51] Langmead B., Salzberg S.L. (2012). Fast gapped-read alignment with Bowtie 2. Nat. Methods.

[52] Roberts A., Pachter L. (2013). Streaming fragment assignment for real-time analysis of sequencing experiments. Nat. Methods.

[53] Dobin A., Davis C.A., Schlesinger F., Drenkow J., Zaleski C., Jha S. (2013). STAR: ultrafast universal RNA-seq aligner. Bioinformatics.

[54] Anders S., Pyl P.T., Huber W. (2015). HTSeq--a Python framework to work with high-throughput sequencing data. Bioinformatics.

[55] Love M.I., Huber W., Anders S. (2014). Moderated estimation of fold change and dispersion for RNA-seq data with DESeq2. Genome Biol..

[56] Afgan E., Baker D., Batut B., van den Beek M., Bouvier D., Cech M. (2018). The Galaxy platform for accessible, reproducible and collaborative biomedical analyses: 2018 update. Nucleic Acids Res..

[57] Goedhart J., Luijsterburg M.S. (2020). VolcaNoseR is a web app for creating, exploring, labeling and sharing volcano plots. Sci. Rep..

[58] Han B.W., Wang W., Zamore P.D., Weng Z. (2015). piPipes: a set of pipelines for piRNA and transposon analysis via small RNA-seq, RNA-seq, degradome- and CAGE-seq, ChIP-seq and genomic DNA sequencing. Bioinformatics.

[59] Ramirez F., Ryan D.P., Gruning B., Bhardwaj V., Kilpert F., Richter A.S. (2016). deepTools2: a next generation web server for deep-sequencing data analysis. Nucleic Acids Res..

[60] Langmead B., Trapnell C., Pop M., Salzberg S.L. (2009). Ultrafast and memory-efficient alignment of short DNA sequences to the human genome. Genome Biol..

[61] Robinson J.T., Thorvaldsdottir H., Winckler W., Guttman M., Lander E.S., Getz G. (2011). Integrative genomics viewer. Nat. Biotechnol..

[62] Rosenkranz D., Han C.T., Roovers E.F., Zischler H., Ketting R.F. (2015). Piwi proteins and piRNAs in mammalian oocytes and early embryos: from sample to sequence. Genom. Data.

