# Supplementary material for: Drosophila Modulo is essential for transposon silencing and developmental robustness
Source: J Biol Chem. 2025 Jan 22;301(3):108210. doi: 10.1016/j.jbc.2025.108210 (PMC11879677; doi:10.1016/j.jbc.2025.108210)
Supplement: Supporting information [file mmc1.pdf]

Supplementary Materials for

Drosophila Modulo is Essential for Transposon Silencing and Developmental Robustness

Rasesh Y. Parikh *et al.*

*Corresponding author. Email: gangaraj@musc.edu

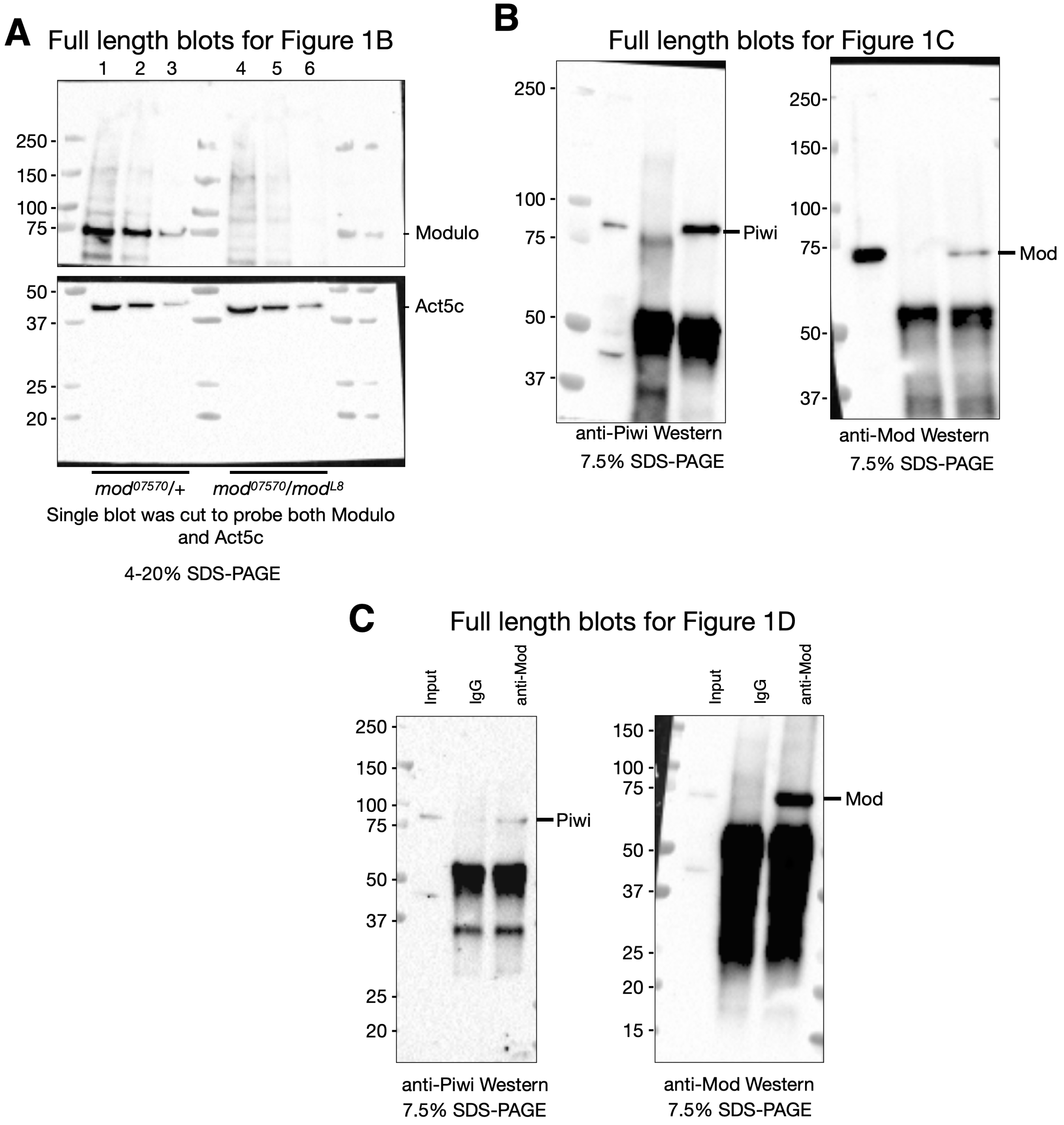

Figure S1: A-C. Full-length blots for the indicated main figures.

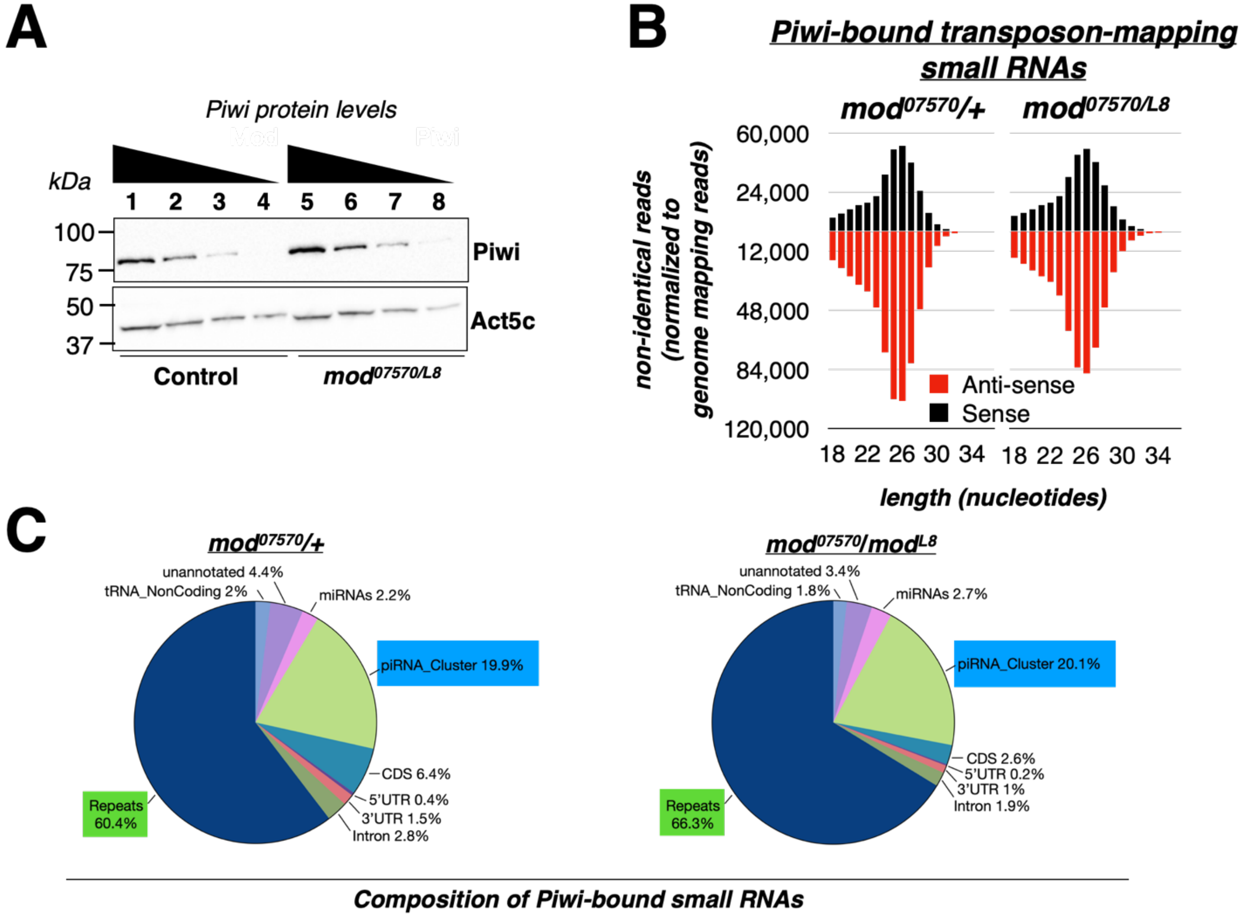

Figure S2: Effect of *mod* mutation on Piwi function. **A)** Immunoblot analysis showing the levels of Piwi protein in *Oregon-R* (control) and *mod*$^{07570/L8}$ ovaries. Three-fold serial dilutions were used. **B)** A column plot showing the size distribution of transposon-mapping sense and anti-sense small RNAs bound to Piwi in *mod*^{07570}/+ and *mod*$^{07570/L8}$ ovaries. **C)** Pie charts displaying the composition of Piwi-bound piRNAs in *mod*^{07570}/+ and *mod*$^{07570/L8}$ ovaries.

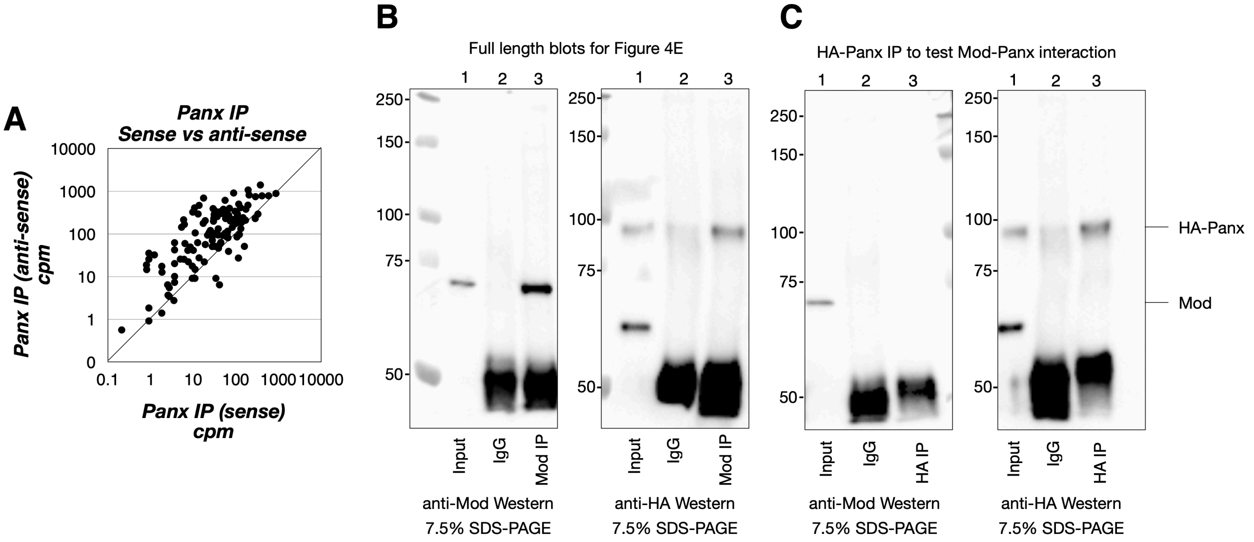

Figure S3: Panx interaction with target transposon RNAs and Mod. A) XY scatter plot showing the level of sense and anti-sense transposon-mapping small RNAs bound to Panx. **B)** Full-length blot for the figure 4E. **C)** Immunoblot analysis testing Panx and Mod interaction after pulling down Panx via its HA epitope tag.

Supplementary Table 1

Antibodies used in this study

**Antibodies**	**Source**	**Application (s)**
Rabbit anti-H3K9me3	Abcam, ab8898	ChIP- 2 μg per IP
Mouse anti-Piwi	Haifan Lin lab	WB- 1:3000; IP- 1:1000; IF-1:500
Mouse anti- Fibrillarin	SantaCruz, Sc 166021	IF- 1:1000
Rabbit anti-beta Actin	Cell signaling, 4970	WB- 1:1000
Mouse anti-Panx	Julius Brennecke lab	WB- 1:50; IP- 50μl per IP
Rabbit anti-HA	Cell Signaling, 3724	WB- 1:1000, IP 1:50
Rabbit anti-Modulo	This study	WB- 1:1000, IF 1:250

Supplementary Table 2

Primers used for ChIP-qPCR (5´-3´) in Fig. 3C

rp49- F	TCCGCCCAGCATACAGGC	rp49-R	CAATCCTCGTTGGCACTCACC
gtwin-F	TTCGCACAAGCGATGATAAG	gtwin-R	GATTGTTGTACGGCGACCTT
gypsy-F	GTTCATACCCTTGGTAGTAGC	gypsy-R	CAACTTACGCATATGTGAGT
diver-F	GGCACCACATAGACACATCG	diver-R	GTGGTTTGCATAGCCAGGAT
